# Sonographic evaluation of spleen size in apparently healthy children in north-west Ethiopia, 2020: time to define splenomegaly

**DOI:** 10.1186/s12887-021-02792-z

**Published:** 2021-07-16

**Authors:** Binalfew Tsehay, Dessalegn Shitie, Abebe Afenigus, Mustofa Essa

**Affiliations:** 1 Department of Biomedical Sciences, Debre Markos University, Debre Markos, Ethiopia; 2 Department of Nursing, Debre Markos University, Debre Markos, Ethiopia; 3 Department of Radiology, Debre Markos University, Debre Markos, Ethiopia

**Keywords:** Reference interval, Sonography, Spleen

## Abstract

**Background:**

Assessment of spleen size is an important part of the clinical skills of medical students and physicians. Many diseases can affect the size of the aforementioned organ, ranging from infective processes to malignant disorders. However, to detect changes, prior knowledge of the actual normal size of these viscera is required in the population being studied. Establishing a customized chart and curve for a specific population of the same sociodemographic characteristics enables a better interpretation of sonographic assessments.

**Methods:**

A hospital-based cross-sectional study design was conducted among 403 children in primary and referral hospitals of the east and west Gojjam zone. Data were collected using a structured questionnaire, physical examination, and ultrasound. The collected data were entered into Epi Data version 3.1 and exports to SPSS version 24 for analysis. Descriptive data were analyzed using descriptive statistics. A Pearson product-moment correlation was run to determine the relationship between age, anthropometric measurements of children, and ultrasound measurements of the spleen. Reference intervals were established using non-parametric reference limits (2.5th -97.5th ) and (5th – 97th ) percentiles by MedCalc software version 20.0.3.

**Results:**

Four hundred three children aged from 7 to 15 years were included in this study. The mean sonographic longitudinal (length), anteroposterior(depth) and transverse (width) dimension of the spleen was, (8.24 ± 1.26 cm), (3.98 ± 0.57 cm), and (4.26 ± 0.59 cm) respectively. The mean volume of the spleen was 75.04 ± 23.92 cm^3^. The height and body surface area of children were best correlated with sonographic dimensions of the spleen. Reference intervals were established using height, age, and body surface area specific for clinically practical dimensions of the spleen.

**Conclusions:**

According to this study, the children are considered as having enlarged longitudinal dimension of the spleen(splenomegaly) if he or she has a size above 97.5th percentile based on their respective height.

## Background

Assessment of spleen size is an important part of the clinical skills of medical students and physicians, and determination of spleen span is essential. Many diseases can affect the spleen size, ranging from infective processes to malignant disorders. However, to detect changes, prior knowledge of the actual normal size of these viscera is required in the population being studied [1]. We can evaluate the size of these visceral organs by clinical and medical imaging techniques.

The clinical assessment of spleen size remains an important part of a physical examination, and knowledge of its normal values at different ages is essential in children and adolescents. This procedure is usually the first step in detecting the abnormal size of the spleen [2]. The spleen is palpable only when it is two to three times its normal size, although it may be palpable in 10 % of healthy children and 15 % of neonates [3]. However, the clinical examination has been established to be often inaccurate in detecting especially small increases in size [4].

Medical imaging techniques permit the observation of anatomical structures in living people and the study of their movements in normal and abnormal activities. Being able to identify normal structures on radiographs makes it easier to recognize the changes caused by disease and injury [5].

However, routine computed tomography and magnetic resonance imaging for the diagnosis and serial follow-up of patients for suspected enlargement of the spleen is difficult to justify because of the radiation exposure, the cost, and limited availability in many areas of the world, particularly in developing countries. So, ultrasonography is an established, safe, fast, and reliable method for the measurement of spleen sizes [6, 7]. In adults, ultrasound indicators of moderate splenomegaly include an anteroposterior diameter greater than two-thirds of the distance between the anterior and posterior abdominal wall, with a craniocaudal length exceeding the upper limit of 11–14 cm [8]. An interpolar diameter greater than 20 cm was considered massive splenomegaly [9].

Unfortunately, there is still no consensus on how to define splenomegaly in pediatric patients. The evaluation is challenging because the normal size varies because of nutritional factors, body habitus, geographical location, physical activities, genetic differences, race, and ethnicity [10–13].

The normal limit of the size of visceral organs according to age, body parameters have not been specified in northwest Ethiopia for school-age children. Hence, our currently used nomograms in primary and referral hospitals are based on the western database, which might lead to under or overestimation of the size of the spleen.

This study aimed to assess and document the splenic sizes in apparently healthy school-age children in an Ethiopian population in the Amhara region and thereby serve as a baseline for comparison in cases of splenomegaly using transabdominal sonography. In addition to this, we evaluated the relationship between splenic measurements, chronological and auxological data, and body proportions.

## Methods

### Study design, setting, and period

The institutional-based cross-sectional study design was conducted among 403 school-age children, from December 2019-June 2020 in Debre Markos referral hospital, Finote Selam general hospital and Bichena primary hospitals of east and west Gojjam zone, northwest Amhara.

### Study participants

All apparently healthy children of 7 to 15 years that came to the hospitals’ pediatrics departments during study periods for a follow-up examination for mildly treated conditions; or who were examined because of problems unrelated to the spleen or a routine check-up were included in the study. However, in children who did not have normal height and weight curves, a history and physical examination finding of oncologic and hematologic disorders or infectious causes of splenic enlargement or splenic trauma, and the accidental discovery of one or more accessory spleens were excluded.

### Sample size and sampling procedures

We calculated the sample size using online sample size calculators for designing clinical research developed by the University of California San Francisco (UCSF) clinical research program; assuming a power of 80 %, correlation coefficient of 0.145 of age, and splenic length among female subjects [14], the significance level of 95 %, and none response rate of 10 %. The computed sample size was 403.

The standard normal deviate for $\mathrm\alpha\;=\;{\mathrm Z}_{\mathrm\alpha}\;=\;1.96$

The standard normal deviate for $\mathrm\beta\;=\;{\mathrm Z}_{\mathrm\beta}\;=\;0.842$  

$$\mathrm C\;=\;0.5\;\ast\;\ln\;\lbrack\;(1+\mathrm r)/\;(1-\mathrm r)\rbrack\;=\;0.147$$

$$\mathrm{Total}\;\mathrm{sample}\;\mathrm{size}\;=\;\mathrm N\;=\;{\lbrack({\mathrm Z}_{\mathrm\alpha}+{\mathrm Z}_{\mathrm\beta})/\mathrm C\rbrack}^2\;+\;3\;=\;366$$

By considering none response rate of 10 % in 37 samples, the total sample size of the study is 403. Assuming the heterogeneity nature of the population, we conducted a purposive sampling technique.

### Data collection procedure and quality control

Sociodemographic characteristics of the study participants were collected using a structured interviewer-administered questionnaire. Anthropometric measurements of the participants, including height (cm), weight (kg), waist circumference (cm), body mass index (BMI), xipho-pubic distance (cm), and abdominal volume, were rigorously evaluated. Height was measured by stadiometer and weight with a standard beam balance scale (digital). Waist circumference was got at the midpoint between the lowest rib and the iliac crest using a tape meter. Xiphopubic distance was measured from the inferior border surface landmark of the xiphoid bone to the superior border of the symphysis pubis. We calculated BMI as body weight (kilograms) divided by body height (meters) squared. Body surface area (BSA) was calculated according to the Mosteller formula: BSA (m2) = square root of ([Height (cm) x Weight (kg)]/ 3600) [15]. The abdominal volume was computed according to the standard formula: (waist Circumference/6.28)^2^ *xipho-pubic distance*3.14 [16]. All anthropometric measurements were taken by three trained medical interns who were in pediatrics attachment.

Sonographic evaluation: All children were asked to lie down in a supine position or a lateral position to get optimal images. We measured: longitudinal dimension: between the highest superior-medial and the lower inferior-lateral points of the spleen; anteroposterior dimension: between the anterior and posterior surfaces; transverse dimension: between the hilum and the superior-lateral edge of the spleen. Scanning was conducted three times, and the average dimension was taken. The volume of the spleen was calculated using the prolated ellipsoid formula (Length *Width*Thickness* 0.523) [17].

To maintain the quality of the research, the data collectors were trained. There was close monitoring and supervision during the data collection period.

### Variables of the study

The dependent variables were sonographic dimensions (longitudinal, anteroposterior, and transverse dimensions and the volume) of the spleen. Whereas socio-demographic; height, weight, waist circumference, body mass index (BMI), body surface area, abdominal volume, and xipho-pubic distances of children were independent variables.

### Definition of variables

Apparently healthy children: refers to the absence of disease of the spleen based on clinical signs and symptoms, normally assessed by history, physical evaluation, and routine laboratory methods available in the hospitals (like for malaria, typhus, and typhoid).

### Data processing and analysis

We used EPI-Data Version 3.1 for data entry and SPSS Version 24 and mainly MedCalc version 20.0.3 for analysis. Descriptive statistics for continuous variables were described using the measure of central tendency (mean) dispersion (standard deviation). A one-way ANOVA was conducted to compare the means of anthropometric measurements among children of each age group. A Pearson product-moment correlation was run to determine the relationship between age, weight, height, waist circumference, xiphopubic distance, body surface area, abdominal volume, and sonographic measurements of the spleen. Pediatric non-parametric reference limits (2.5th —97.5th and 3rd – 97th percentiles) for spleen measures are reported. Model-based specific reference intervals were computed with age, height, and body surface area modeled as fractional polynomials. Transformation of variables was conducted before model fitting and then plotted against height body surface area and age.

## Results

### Anthropometric assessments of children

A total of four hundred three apparently healthy children were enrolled in this study. A one-way ANOVA was conducted to compare the means of anthropometric measurements among children of each age group. There was a statistically significant difference between groups ANOVA (F (8,394) = 191.66, *p* < 0.001) for height; F (8,394) = 73.06, *p* < 0.001) for weight; F (8,394) = 24.97, *p* < 0.001) for waist circumference; F (8,394) = 101.95, *p* < 0.001) for xiphopubic distance; F (8,394) = 103.61, *p* < 0.001) for body surface area, and F (8,394) = 44.53, *p* < 0.001) for abdominal volume (Table 1).

**Table 1:** Descriptive analysis of anthropometric measurements of children based on age (*n* = 403)

Age	N	Height(cm)		Weight (kg)		Waist circumference (cm)		xiphopubic distance(cm)		body surface area (m2)		abdominal volume(cm3)	
		Mean	SD	Mean	SD	Mean	SD	Mean	SD	Mean	SD	Mean	SD
7	60	118.9	6.2	25.3	4.0	52.5	5.2	23.6	2.2	0.9	0.1	5233.0	1232.2
8	40	119.6	5.4	26.1	4.1	53.4	5.0	25.5	1.1	0.9	0.1	5824.7	1001.2
9	52	133.5	8.4	35.5	8.5	57.0	4.9	25.5	2.1	1.1	0.2	6585.0	958.7
10	76	134.8	7.5	36.4	7.3	56.8	3.6	28	2	1.2	0.1	7233.0	1156.7
11	16	140.5	0.5	49.8	0.9	62.8	0.4	27.3	0.4	1.4	-	8541.4	16.7
12	48	137.2	7.5	33.8	7.8	56.3	3.3	28.0	1.0	1.1	0.2	7075.7	870.9
13	34	153	1.1	44.8	5.5	61.6	1.7	31.4	1.0	1.4	0.1	9509.0	694.5
14	24	153.3	4.5	45.5	1.7	57.7	2.3	32.8	1.5	1.4	-	8690.2	585.4
15	53	150.4	2.6	44.0	5.9	59.1	4.2	30.8	3.1	1.4	0.1	7871.0	2563.3
Total	403	136.0	13.0	36.1	9.6	56.8	4.9	27.7	3.4	1.2	0.2	7107.8	1790.7

### Sonographic evaluation of spleen and anthropometric measurements (body parameters)

The mean sonographic longitudinal (length), anteroposterior(depth) and transverse (width) dimension of the spleen was, (8.24 ± 1.26 cm), (3.98 ± 0.57 cm), and (4.26 ± 0.59 cm) respectively. The mean volume of the spleen was 75.04 ± 23.92cm^3^. The longitudinal dimension of the spleen was moderately correlated with height (*r* =. 384, *n* = 403, *p* < 0.001). The anteroposterior dimension (thickness) of the spleen was best correlated with the weight (*r* = 0.475, *n* = 403, *p* < 0.001) and body surface area (*r* = 0.493, *n* = 403, *p* < 0.001) of the children. Similarly, the volume of spleen showed moderate correlation with the weight (*r* = 0.464, *n* = 403, *p* < 0.001) and body surface area (*r* = 0.479, *n* = 403, *p* < 0.001) of the children but best predicted by their age. However transverse diameter of the spleen showed a weak correlation with body parameters (Table 2).

**Table 2:** Pearson correlation matrix of spleen sonographic dimensions and anthropometric profiles of children (*n* = 403)

Variables		Longitudinal dimension of spleen(cm)	Anteroposterior dimension of spleen(cm)	Transverse dimension of spleen(cm)	The volume of spleen (cm3)
Age in years	Pearson Correlation	0.344^**^	0.358^**^	0.208^**^	0.402^**^
	Sig. (2-tailed)	0.000	0.000	0.000	0.000
Height(cm)	Pearson Correlation	0.384^**^	0.455^**^	0.123^*^	0.439^**^
	Sig. (2-tailed)	0.000	0.000	0.013	0.000
Weight(kg)	Pearson Correlation	0.270^**^	0.475^**^	0.242^**^	0.464^**^
	Sig. (2-tailed)	0.000	0.000	0.000	0.000
Waist circumference(cm)	Pearson Correlation	0.230^**^	0.441^**^	0.132^**^	0.392^**^
	Sig. (2-tailed)	0.000	0.000	0.008	0.000
Xiphopubic distance(cm)	Pearson Correlation	0.245^**^	0.311^**^	0.022	0.263^**^
	Sig. (2-tailed)	0.000	0.000	0.663	0.000
Body mass index	Pearson Correlation	0.010	0.148^**^	0.108^*^	0.159^**^
	Sig. (2-tailed)	0.837	0.003	0.030	0.001
Body surface area	Pearson Correlation	0.323^**^	0.493^**^	0.210^**^	0.479^**^
	Sig. (2-tailed)	0.000	0.000	0.000	0.000
Abdominal volume	Pearson Correlation	0.262^**^	0.400^**^	0.098^*^	0.368^**^
	Sig. (2-tailed)	0.000	0.000	0.050	0.000

*Correlation is significant at the 0.05 level (2-tailed). **Correlation is significant at the 0.01 level (2-tailed)

### Reference intervals for dimensions of spleen and body parameters

**Fig. 1:**
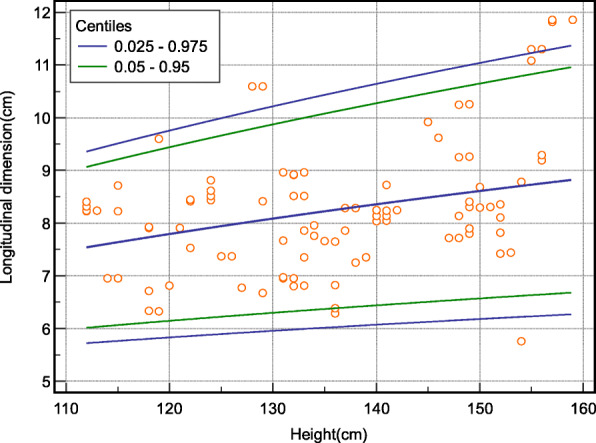
Percentile curves of the model-based reference limits (2.5th -97th and 5th -95th ) for the longitudinal dimension of the spleen

**Fig. 2:**
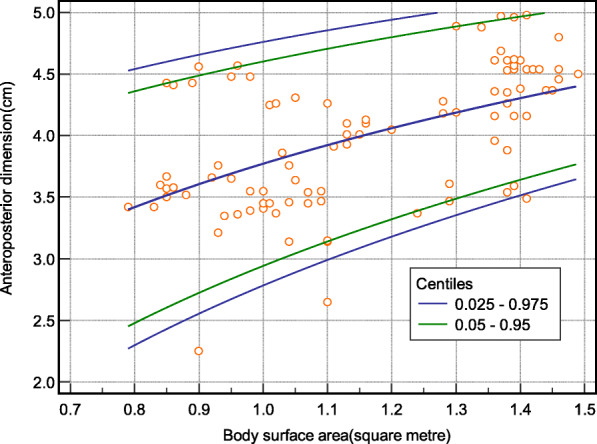
Percentile curves of the model-based reference limits (2.5th -97th and 5th -95th ) for the anteroposterior dimension of the spleen

**Fig. 3:**
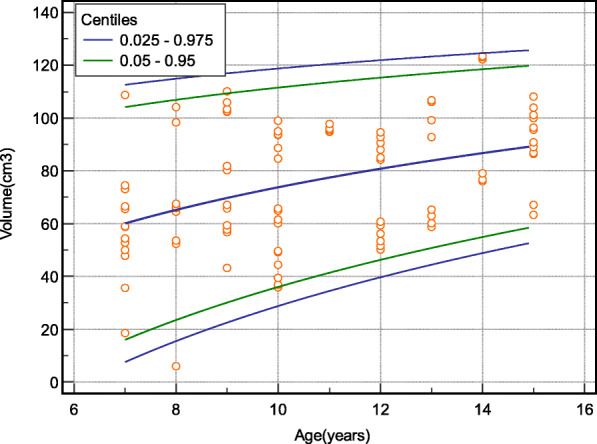
Percentile curves of the model-based reference limits (2.5th -97th and 5th -95th ) for the volume of the spleen

Model-based body parameter-specific reference intervals were computed, using MedCalc version 20.0.3. We established smoothened height for the longitudinal dimension of the spleen (Table 3; Fig. 1); body surface area for the anteroposterior dimension (Table 4; Fig. 2), and age for the volume of the spleen (Table 5; Fig. 3) using non-parametric reference limits (2.5th -97.5th ) and 5th – 97th percentiles).

**Table 3:** Limit of a longitudinal dimension of spleen according to the height of children using non-parametric reference limits (2.5th -97.5th ) and 5th – 97th percentiles) (*n* = 403)

Variable	Centiles of the longitudinal dimension			
Height	**0.025**	**0.05**	**0.95**	**0.975**
115	5.8	6.1	9.4	9.76
120	5.8	6.1	9.7	9.99
125	5.9	6.2	9.9	10.2
130	6	6.3	10.1	10.4
135	6	6.4	10.3	10.6
140	6.1	6.4	10.5	10.8
145	6.1	6.5	10.7	11
150	6.2	6.6	10.8	11.2
155	6.2	6.6	9.4	9.76

**Table 4:** Limit of the anteroposterior dimension of the spleen(cm) according to body surface area of the children using non-parametric reference limits (2.5th -97.5th ) and 5th – 97th percentiles) (*n* = 403)

Variable	Centiles of Anteroposterior dimension of spleen			
Body surface area (square metre)	**0.025**	**0.05**	**0.95**	**0.975**
0.8	2.3	2.5	4.4	4.5
0.9	2.6	2.7	4.5	4.7
1.0	2.8	2.9	4.6	4.8
1.1	3.0	3.1	4.7	4.9
1.2	3.2	3.3	4.8	4.9
1.3	3.4	3.5	4.9	5.0
1.4	3.5	3.6	5.0	5.1

**Table 5:** limit of the volume of spleen according to the age of the children using non-parametric reference limits (2.5th -97.5th ) and 5th – 97th percentiles) (*n* = 403)

Variable	Centiles of Volume of the spleen			
Age	**0.025**	**0.05**	**0.95**	**0.975**
7	7.57	16	104.2	112.6
8	15.5	23.5	106.9	114.9
9	22.5	30.1	109.4	117
10	28.8	36	111.6	118.8
11	34.5	41.4	113.5	120.4
12	39.7	46.3	115.3	121.9
13	44.4	50.8	117	123.3
14	48.8	54.9	118.5	124.6

### When to say splenomegaly?

According to our study, the children are considered as having enlarged longitudinal dimension of the spleen (splenomegaly) if he or she has a size above 97.5th percentile based on the respective height.

## Discussion

In the literature, there are few detailed studies to interpret the spleen dimensions in school-aged children in Africa and Ethiopia. Sonographic determination of pathologic changes in the size of the spleen necessitates knowing the normal ranges of its measurements especially concerning anthropometric assessments in school-age children. The morphology of the spleen varies from person to person. During the maturation process from infancy through adolescence, the growth of the spleen shows a high correlation with gains in height, weight, and body surface area [6, 18].

To the best of our knowledge, our study, which aimed to investigate the normal limits of the spleen of school-aged children, comprises one of the largest series in the literature from Ethiopia, including the age group of 7 to 15 years.

There is no agreement on which anthropometric measurements are generally acceptable for exploring the normal limit of organ measurements [6, 19–21] as these parameters might varies due to nutritional factors, body habitus, geographical location, physical activities, genetic differences, race, and ethnicity. Previous studies showed that the longitudinal dimensions of the spleen, were best correlated with body parameters [6, 21–30]. This is in accordance with the results of our study. The height, weight, and body surface area of the children were best correlated with the dimensions of the spleen.

Assessment of longitudinal dimensions (length) of the spleen is more practical either by palpation and percussion or using ultrasound. The estimation of spleen width and volume is less solid and questionable in diagnosing splenomegaly [31]. In this study, the height of children was best correlated with the longitudinal dimensions of the spleen hence reference intervals and curves were established using the height of the children. The transverse dimension of the spleen showed a weak correlation with the body parameters. This is supported by similar studies in different countries [6, 32]. However, others reported that weight was best correlated with longitudinal dimensions of the spleen [24, 33]. These differences might be because of variations in the race or different ethnic origins, nutritional factors, body habitus, and geographical location.

In most studies, sizes between the 5th and 95th percentiles were the accepted normal limits [33, 34]. This practice results in approximately 10 % of children with normal visceral organs falling outside these limits [21]. We preferred to define the lowermost and uppermost dimensions of the spleen to best correlated anthropometric measurements using the 2.5th and 97.5th percentile values in addition to the 5th and 95th percentile values respectively as a guide.

## Conclusions

The normal limits of the spleen are important parameters during a sonographic examination. Reference intervals were established based on the best-correlated and predictor anthropometric measurements. Children with the longitudinal dimension of spleen above 97.5th percentile values with their specific height are considered as having splenomegaly. We hope this study contributes to daily practice in radiology clinics to interpret the normal sizes of the spleen of school-aged children in northwest Ethiopia.

### Limitations

Calibration of measuring tools in the hospitals were not conducted frequently.

## Data Availability

The datasets used and/or analyzed during the current study are available from the corresponding author on reasonable request.
